# Signaling Flux Redistribution at Toll-Like Receptor Pathway Junctions

**DOI:** 10.1371/journal.pone.0003430

**Published:** 2008-10-17

**Authors:** Kumar Selvarajoo, Yasunari Takada, Jin Gohda, Mohamed Helmy, Shizuo Akira, Masaru Tomita, Masa Tsuchiya, Jun-ichiro Inoue, Koichi Matsuo

**Affiliations:** 1 Institute for Advanced Biosciences, Keio University, Tsuruoka, Japan; 2 Department of Microbiology and Immunology, School of Medicine, Keio University, Tokyo, Japan; 3 Division of Cellular and Molecular Biology, Institute of Medical Science, The University of Tokyo, Tokyo, Japan; 4 Department of Host Defense, Research Institute for Microbial Diseases, Osaka University, Osaka, Japan; University of Miami, United States of America

## Abstract

Various receptors on cell surface recognize specific extracellular molecules and trigger signal transduction altering gene expression in the nucleus. Gain or loss-of-function mutations of one molecule have shown to affect alternative signaling pathways with a poorly understood mechanism. In Toll-like receptor (TLR) 4 signaling, which branches into MyD88- and TRAM-dependent pathways upon lipopolysaccharide (LPS) stimulation, we investigated the gain or loss-of-function mutations of MyD88. We predict, using a computational model built on the perturbation-response approach and the law of mass conservation, that removal and addition of MyD88 in TLR4 activation, enhances and impairs, respectively, the alternative TRAM-dependent pathway through *signaling flux redistribution* (*SFR*) at pathway branches. To verify *SFR*, we treated MyD88-deficient macrophages with LPS and observed enhancement of TRAM-dependent pathway based on increased IRF3 phosphorylation and induction of *Cxcl10* and *Ifit2*. Furthermore, increasing the amount of MyD88 in cultured cells showed decreased TRAM binding to TLR4. Investigating another TLR4 pathway junction, from TRIF to TRAF6, RIP1 and TBK1, the removal of MyD88-dependent TRAF6 increased expression of TRAM-dependent *Cxcl10* and *Ifit2*. Thus, we demonstrate that *SFR* is a novel mechanism for enhanced activation of alternative pathways when molecules at pathway junctions are removed. Our data suggest that *SFR* may enlighten hitherto unexplainable intracellular signaling alterations in genetic diseases where gain or loss-of-function mutations are observed.

## Introduction

The TLR4 is a transmembrane receptor for LPS, found on the outer membrane of Gram-negative bacteria [1], [2]. Upon LPS binding, TLR4 triggers two major intracellular pathways, the MyD88-dependent and –independent (TRAM-dependent) pathways [3]. The MyD88-dependent pathway mainly induces proinflammatory cytokines such as TNFα, IL-6, and SOCS3 through activation of p38, ERK, JNK and NF-κB. The TRAM-dependent pathway, on the other hand, predominantly induces type I interferons (IFNs) and chemokines such as IP-10 (encoded by *Cxcl10*) and interferon (IFN)-induced proteins through activation of interferon regulatory factor (IRF) 3 or 7 and NF-κB [4]. Thus, both pathways complement each other in the production of pro-inflammatory mediators.

Previous studies on the TLR4 pathway have largely ignored the effects on TRAM-dependent pathways in MyD88 or TRAF6 knockout (KO) mice [5]–[8]. Here we developed computational model built using perturbation-response [9], [10] approach and analyzed the dynamic behavior of the TLR4 pathway combining simulations with molecular experiments. From *in silico* MyD88 KO or TRAF6 KO, we simulated enhanced activation of alternative TRAM-dependent pathway through the re-channelling of signal transduction or *SFR*, which occurs when molecules at pathway junction are removed. These findings were validated through *in vivo* and *in vitro* experiments. Thus, *SFR* may explain the mechanistic basis for unexpected alterations in cellular signaling, for example, due to gain or loss-of-function mutations found in human diseases [11], [12].

## Results and Discussion

### Simulating the MyD88-dependent pathway

**Figure 1:**
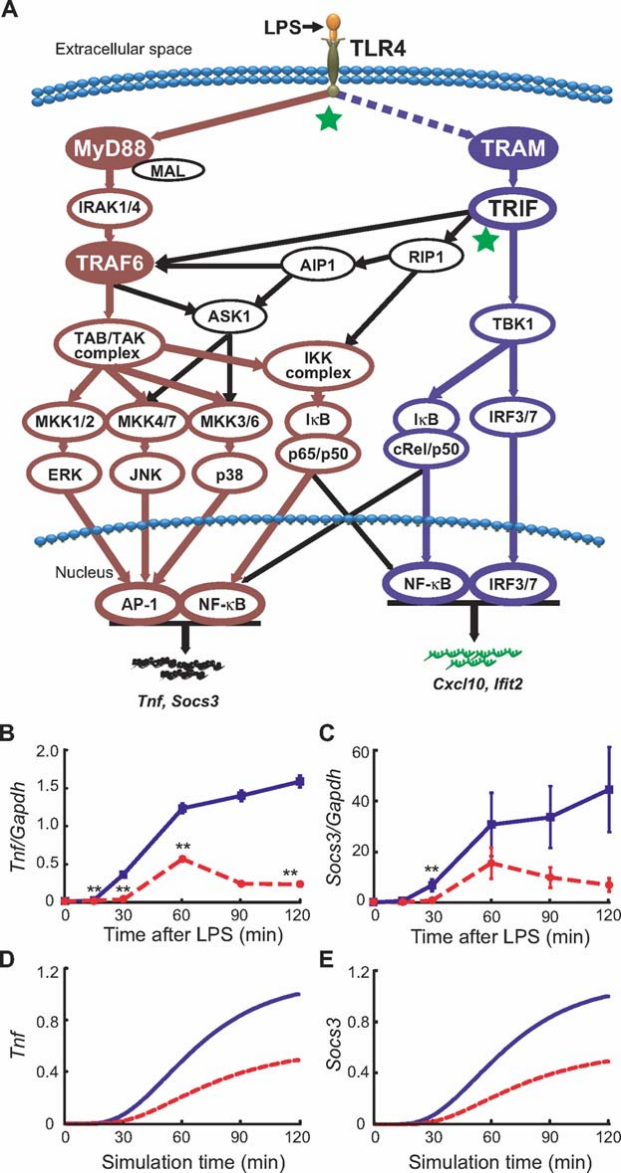
Schematic presentation of TLR4 pathway and temporal profiles of experimental and simulated *Tnf* and *Socs3*. (A) Following LPS stimulation, TLR4 signaling bifurcates into MyD88-dependent (brown) and TRAM-dependent (blue) pathways, which activates target genes such as *Tnf* and *Socs3*, and *Cxcl10* and *Ifit2*, respectively. Black arrows indicate cross talks between MyD88-dependent and TRAM-dependent pathways. The two junctions analyzed in this study are indicated by stars (TLR4 to MyD88/TRAM and TRIF to TRAF6/RIP1/TBK1). Dashed line between TLR4 and TRAM represents indirect activation of TRAM for its recruitment to the TLR4 ([9] and supplementary Table SI). (B) *Tnf* and (C) *Socs3 in vivo* mRNA levels after LPS treatment in wildtype (blue) and MyD88 KO (red, dotted) macrophages measured by qRT-PCR. Values are an average of six independent cultures and shown as means±SEM. *In silico* simulated expression (arbitrary units) of (D) *Tnf* and (E) *Socs3* in the presence (blue) and absence (red, dotted) of MyD88 upon TLR4 activation. **p<0.01 vs. wildtype.

We updated our previous TLR4 model [9] incorporating several new features into the topology; i) crosstalk mechanisms from TRIF to TRAF6 and TRIF to RIP1 for NF-κB (p65/p50) and MAP kinases activation, ii) the addition of MKK1/2-ERK-AP-1 activation pathway, and iii) phosphorylation of the IκB/c-Rel/p50 complex by TBK1 leading to NF-κB (c-Rel/p50) activation (Figure 1A and supplementary Table SI). Furthermore, to predict gene transcription we included several intermediate reactions between activation of transcription factors and target mRNA transcription to represent delays potentially due to chromatin remodeling and initiation complex formation [13], [14].

The *in silico* model begins with LPS-activated TLR4, which results in signal flux propagation through the MyD88-dependent and TRAM-dependent pathways (Figure 1A). The perturbation-response approach was used to represent the complex reaction mechanism [10], [15] of the TLR4 pathways. Therefore, each reaction in the signaling pathway is represented with first order kinetics with pulse perturbation given to mass-conserved TLR4 system (Methods and [9], [10], [16]). The parameter values for all reactions, except for the new reactions, were initially obtained from our previous model. Next, we adjusted these values and chose the parameters of the new reactions to fit experimental time-course mRNA profiles of *Tnf* and *Socs3* in wildtype macrophages following LPS stimulation (Figure 1B,C, blue). Indeed, the selected parameters produced simulation that is quantitatively consistent with p38, ERK, JNK, NF-κB (supplementary Figure S1 and [3], [7]), *Tnf* and *Socs3* (Figure 1D,E, blue) of wildtype condition. We then removed MyD88 *in silico* by shutting down the TLR4→MyD88 reaction (Reaction 1 in supplementary Table SI) and simulated delayed and impaired activation of p38, ERK, JNK, NF-κB, *Tnf* and *Socs3* (supplementary Figure S1 and Figure 1D,E, red), which matches experimental observations with MyD88-deficient macrophages upto 60 min (Figure 1B,C, red and [3], [7]). Beyond 60 min, *Tnf* and *Socs3* expression for MyD88-deficient macrophages was downregulated presumably by posttranscriptional regulation [17], [18] which was not considered in our model.

### Simulating the TRAM-dependent pathway

**Figure 2:**
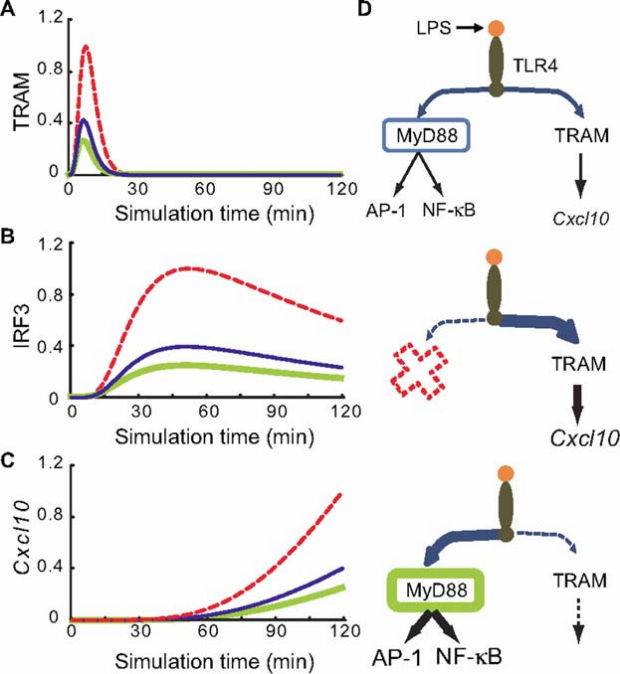
*In silico* simulations of TRAM-dependent pathway molecules upon TLR4 activation. Simulation profiles (arbitrary units) of (A) TRAM activation, (B) IRF3 activation, and (C) *Cxcl10* induction in the wildtype (blue), knockout (red, dotted), and two-fold overexpression (green) of MyD88. (D) Schematic of *SFR*. (Top) Wildtype. Fluxes propagate through both the MyD88-dependent and TRAM-dependent pathways. (Middle) MyD88 KO. More fluxes propagate or *overflows* through the TRAM-dependent pathway resulting in increased *Cxcl10* induction. (Bottom) MyD88 overexpression by two-fold.

Using our model, we next investigated the TRAM-dependent pathway in wildtype and MyD88 KO conditions. From our current knowledge, the removal of MyD88 should not change TRAM-dependent activation. However, *in silico* removal of MyD88 resulted in the increased activation of TRAM-dependent pathway compared to wildtype model, as illustrated by increased activation of TRAM, IRF3 and *Cxcl10* (Figure 2A–C, red). In our *in silico* model, the removal of MyD88 solely abolishes the propagation of signal transduction from TLR4 to MyD88. As a result, interaction between TLR4 and TRAM increases due to the law of mass conservation (see “Signaling Flux Conservation”, Methods).

Alternative to the loss-of-function experiments described so far, we examined the signaling outcome of gain-of-function mutations, which are frequently observed in genetic diseases [11], [12], [19]. To mimic such a scenario, we performed *in silico* overexpression of MyD88 by increasing the rate of TLR4→MyD88 reaction (Reaction 1 in supplementary Table SI) and the simulations resulted in reduced levels of TRAM, IRF3 and *Cxcl10* (Figure 2A–C, green) and increased JNK and NF-κB activities (supplementary Figure S2A,B) in a dose-dependent manner (data not shown). That is, when molecules at pathway junction are decreased and increased the activation of alternative pathways enhances and reduces, respectively. We describe this re-channelling of signal transduction as *signaling flux redistribution* or *SFR* (Figure 2D).

### Validating the occurrence of *SFR*

**Figure 3:**
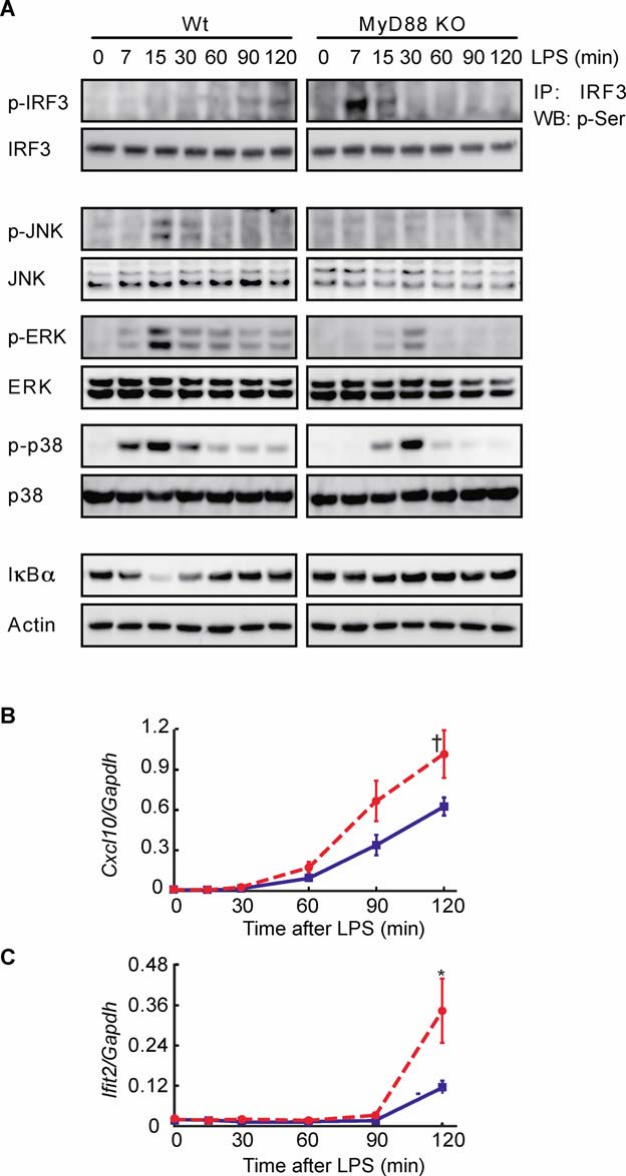
Enhanced TRAM-dependent pathway in the absence of MyD88. Macrophages were treated with LPS (100 ng/ml) for indicated periods. Cell lysates were analyzed for (A) IRF3, JNK, ERK, p38 phosphorylation and NF-κB (degradation of IκBα) using Western blot analysis with Actin as a loading control. (B) *Cxcl10* and (C) *Ifit2* mRNAs levels in wildtype (blue) and MyD88 KO (red, dotted) macrophages using qRT-PCR. Six independent cultures were analyzed and shown as means±SEM. †p = 0.064.

To examine whether *SFR* occurs in actual cells, we prepared macrophages from wildtype and MyD88-deficient mice and measured levels of TRAM-dependent IRF3 phosphorylation, as well as activation of MAP kinases JNK, ERK, p38 and NF-κB (IκBα degradation) after LPS stimulation. In accordance with previous studies [7], [8], MAP kinases and NF-κB activation was impaired and delayed in MyD88-deficient macrophages compared to wildtype macrophages (Figure 3A). Notably, as predicted by our computational model, increased IRF3 phosphorylation and induction of *Cxcl10* and *Ifit2* mRNAs was observed for MyD88-deficient macrophages (Figure 3A–C). We hypothesise the faster experimental kinetics of IRF3 (Figure 3A, top right panel) compared with *in silico* simulation in Figure 2B is a consequence of increased activation of TRAM-dependent pathway in MyD88-deficient macrophages (which requires increase in both *k*_i_ and *S*_i_, Methods). These data support the occurrence of *SFR*.

### Removal and addition of MyD88 in cultured cells reveal competition between MyD88 and TRAM

**Figure 4:**
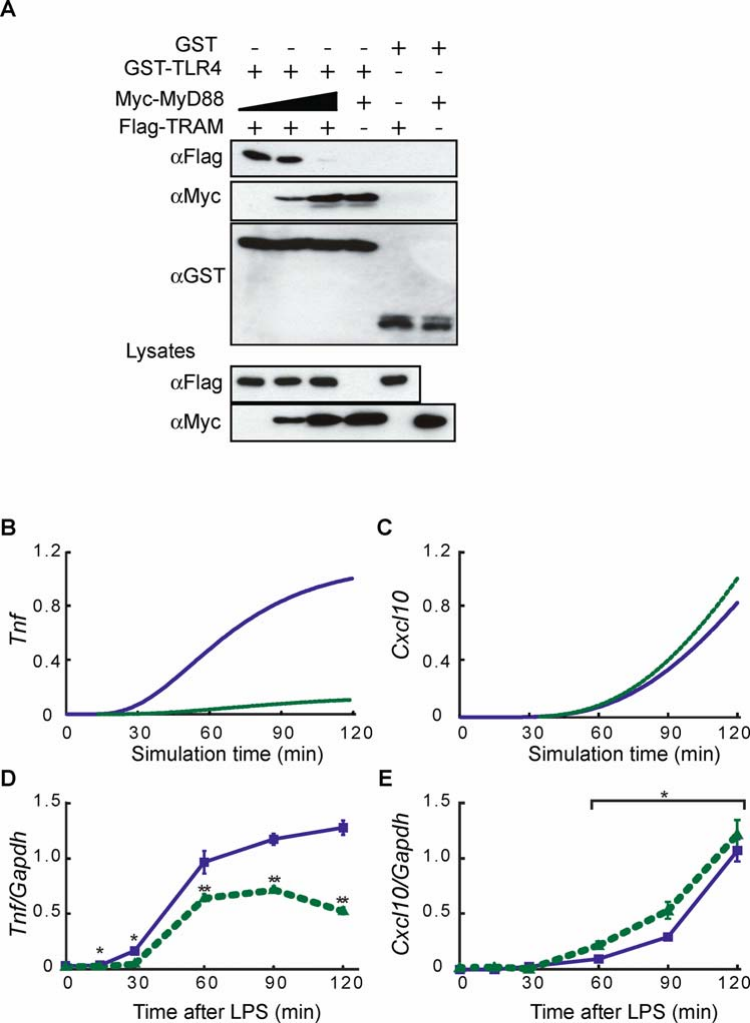
Competition at TLR4 and *SFR* in TRAF6 KO. (A) MyD88 and TRAM compete for TLR4 in GST pull-down assay. GST or the GST-tagged TLR4 were expressed in HEK293T cells with Myc-tagged MyD88 or Flag-tagged TRAM. After GST-pull-down, Western blotting was performed. (B–E) Enhanced TRAM-dependent pathway in the absence of TRAF6. *In silico* expression of (B) *Tnf* and (C) *Cxcl10*, and *in vivo* expression of (D) *Tnf* and (E) *Cxcl10* mRNA in wildtype (blue) and TRAF6 KO (green, dotted) macrophages. Four independent cultures were analyzed. means±SEM. *p<0.05, **p<0.01 vs. wildtype. Paired student's t-test was used for (E).

The TLR4 (TIR domain) carries a multifunctional docking site where MyD88, Mal, TRAM, and TRIF adaptor molecules bind with common specificity [20]. To determine the molecular mechanism for *SFR* in TLR4 signaling, we performed a competition assay in cultured cells by overexpressing TLR4 cytoplasmic tail, MyD88 and TRAM. Figure 4A shows that TRAM interacted with TLR4 cytoplasmic tail in HEK293T cells. When the concentration of MyD88 was increased, MyD88 preferentially competed away TRAM from TLR4 in a dose-dependent manner, indicating that MyD88 and TRAM bind to TLR4 competitively. This is consistent with the prediction of the *in silico* model (Figure 2A), and suggests that increased TRAM binding with TLR4 in the absence of MyD88 is due to loss of competition between MyD88-dependent and TRAM-dependent pathways.

### Enhanced TRAM-dependent pathway in TRAF6 KO

To check whether *SFR* is a general property of signal transduction at pathway junctions, we next analyzed another branch point of the TRAM-dependent component of the TLR4 pathway: TRIF to TBK1, TRAF6 and RIP1 (Figure 1A). Since TBK1 and TRAF6 compete to bind to the N-terminal domain of TRIF [21], we determined the consequence of removing TRAF6 molecules on the TRAM-dependent pathway. We computationally simulated TRAF6 knockout conditions and predicted lower induction of *Tnf*, however, greater induction of *Cxcl10* in the absence of TRAF6 due to increased propagation of signaling flux through the TRIF-TBK1-IRF3 route (Figure 4B,C). We performed parallel bench experiments generating macrophages from wildtype and TRAF6-deficient mice [22] and treated them with LPS. While *Tnf* expression was lower as predicted and previously observed [5], *Cxcl10*, *Ifit1* and *Ifit2* induction was higher in TRAF6-deficient macrophages than in wildtype macrophages (Figure 4D,E and supplementary Figure S3A,B).

### Further insights of *SFR* in the TLR4 pathway

**Figure 5:**
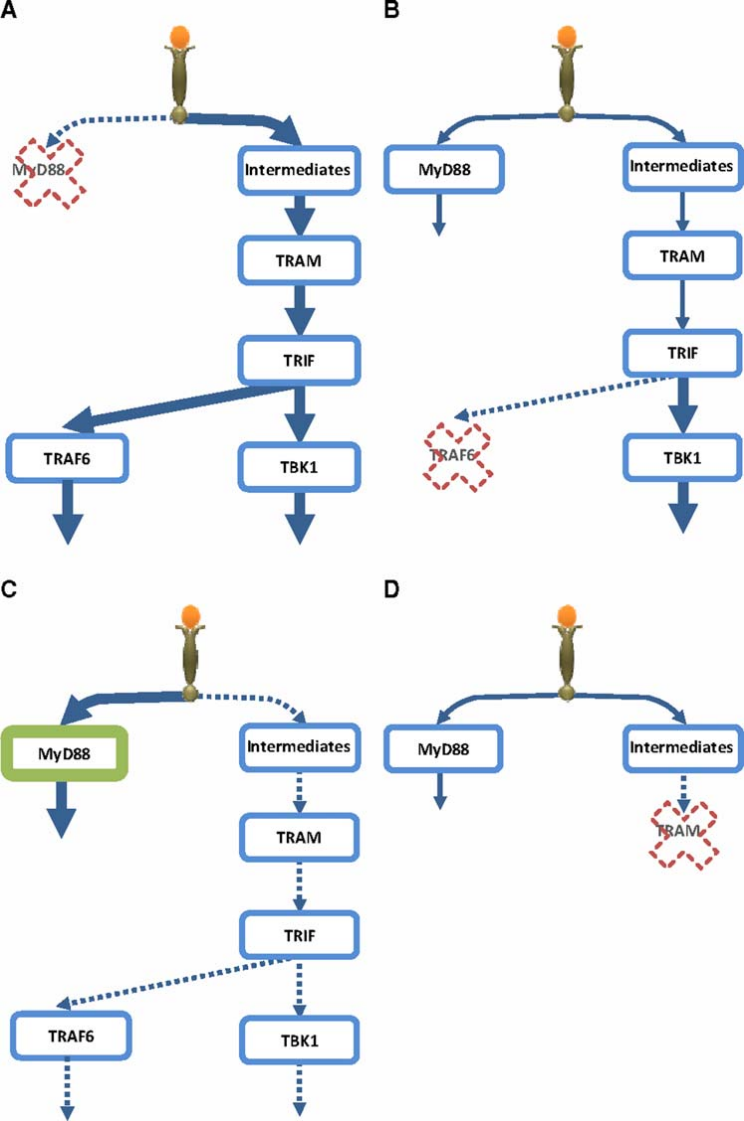
Schematic representation of *Signaling Flux Redistribution* (*SFR*). (A) removal of MyD88 results in enhancement of TRAM-dependant pathway, (B) removal of TRAF6 results in enhancement of TRAM-dependant pathway downstream of TRIF, (C) overexpression of MyD88 downregulates TRAM-dependant pathway, (D) removal of TRAM does not enhance the MyD88-dependant pathway due to upstream intermediates. * signaling molecules/events upstream of TRAM [9], [25], [26].

Since cells are able to execute numerous processes using only a limited set of interaction domains that have flexible binding properties [23], we believe *SFR* at these domains can enhance or impair alternative pathways when a competing molecule such as MyD88 or TRAF6 is removed (Figure 5A,B) or increased (Figure 5C). In addition to TRAF6 and TBK1, RIP1 and RIP3 also compete for TRIF, and binding of RIP3 to TRIF is increased in the absence of RIP1 [24]. *SFR* predicts the enhanced activation of the RIP3-dependent pathway in RIP1-deficient cells.

Our *in vitro* experiments have shown that TRAM and MyD88 compete for TLR4. Using *SFR* on these data would suggest enhancement of MyD88-dependent pathway in the removal of TRAM. Contrary, *in vivo* studies [3] have shown impaired activation of MyD88-dependent pathway; reduced activation of NF-κB, JNK, TNFα and IL-6 in TRAM KO. The absence of *SFR* is due to the fact that *in vivo* additional signaling intermediates act upstream of TRAM ([3], [25], [26] and supplementary Table SI online). Our *in silico* simulation is consistent with the idea that the lack of *SFR* is a result of signaling flux trapped by the intermediates upstream of TRAM (Figure 5D, data not shown). Thus, *SFR* can also be used to determine whether competing molecules *in vitro* are at pathway junctions *in vivo*.

### *SFR* in other pathways

The observation of enhanced alternative pathways when molecules at pathway junction are removed may not be restricted to TLR4 signaling. Recent work on HELA and H460 cells focusing on TRADD and RIP, which binds to intracellular TNFR1, demonstrate that deletion of either molecules result in enhancement of alternative pathways in TNF stimulation [27].

In another study, markedly elevating Ser/Thr phosphorylation in rat hepatoma Fao cells reduced alternative insulin-induced Tyr phosphorylation of IRS-1 and IRS-2, which significantly reduced their ability to interact with the juxtamembrane region of insulin receptor resulting in impaired downstream signal [28]. Reversing these effects by incubating cell extracts with alkaline phosphatase strongly indicated that insulin resistance is associated with enhanced Ser/Thr phosphorylation of IRS-1 and IRS-2.

**Figure 6:**
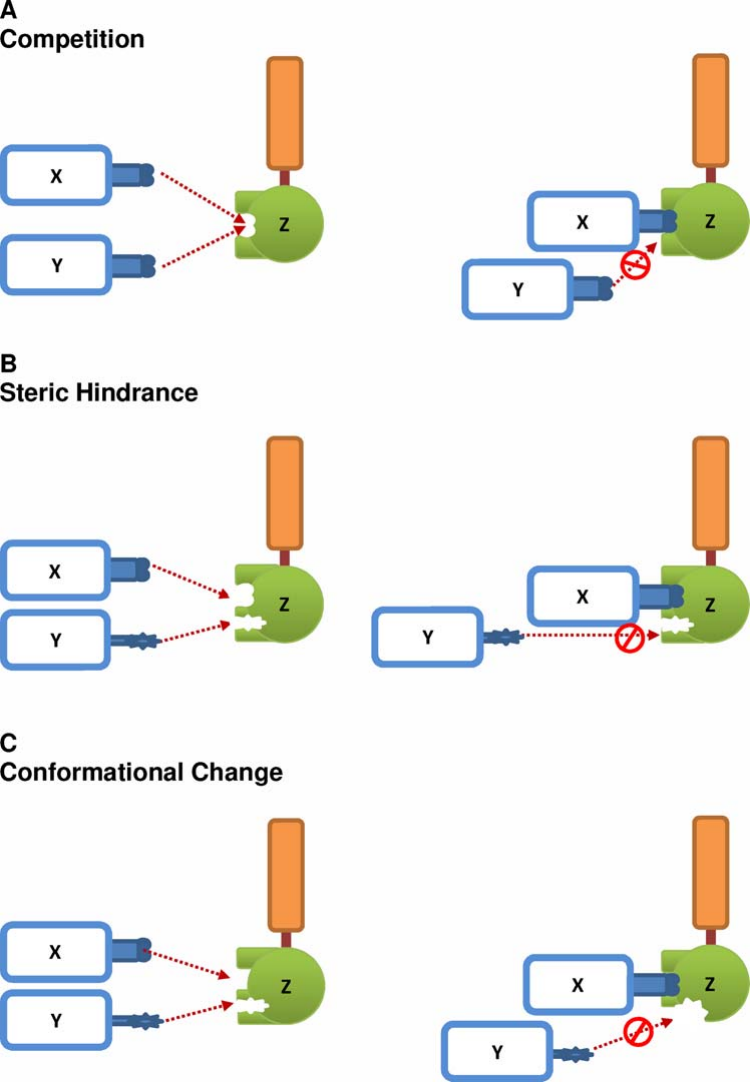
Proposed mechanisms of action for *SFR*. (A) Competition: Molecules X and Y compete to bind with molecule Z. X and Y share binding sites at Z. (B) Steric hindrance: When X binds to Z, the complex prevents the binding of Y to another binding site at Z (C) Conformational change: When X binds to Z, structural changes to Z lowers the affinity of Y binding to Z.

Thus, *SFR* could be a general property occurring at signaling pathway junctions. Although there may be several mechanisms for *SFR* occurrence, we propose three possibilities of action: competition, physical blocking or conformational change (Figure 6).

### Conclusion

In summary, using *in silico* TLR4 model we predicted and experimentally validated the enhancement of TRAM-dependent pathways due to *SFR* in the removal of MyD88 and TRAF6. The enhancement of entire TRAM-dependent pathways in MyD88 KO through *SFR* provides an alternative mechanism that does not require any physical negative crosstalk interaction between the MyD88 and TRAM molecules. *SFR* is, therefore, a novel mechanism for regulating the balance between alternative pathways and can be successfully used to predict the molecular dynamics of entire signaling pathways. Although we have demonstrated *SFR* for molecules with common binding domain, *SFR* might also occur between molecules with different binding domains at pathway junctions from the law of mass flow conservation (Figure 6). Whether *SFR* can be used to understand the enhancement or repression of alternative pathways in genetic diseases where gain or loss-of-function mutations occur remains an interesting topic for investigation.

## Materials and Methods

### Computational Model

The basic principle behind our approach is to induce a controlled perturbation of input reaction species of a system (TLR4), and monitor the response of the concentration/activation levels of other output species (e.g. JNK, NF-κB, IRF3 activation) from steady-state. To illustrate, let us perturb a stable biological network consisting of *n* species from reference (stable) steady-state. The deterministic kinetic evolution equation is
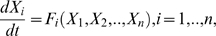
(1)where the corresponding vector form of Eq. 1 is 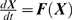 and ***F*** is a vector of any function (non-linear in general) of the species vectors ***X*** = (*X_1_*, *X_2_*, ‥, *Xn*) representing concentration or activation levels. The response to perturbation can be written by ***X*** = ***X***_0_+δ***X***, where *X_0_* is reference steady-state vector and δ*X* is relative response from the steady-states (δ***X*** *_t = 0_* = **0**).

The use of small perturbation around steady-state leads to important simplification to the evolution equation (Eq. 1), which can be highly non-linear, resulting in the approximation of the first-order term, in vector form is 
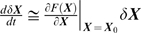
, noting that zeroth order term ***F***(***X***_0_) = 0 at the steady-state ***X*** **_0_** and the Jacobian matrix or linear stability matrix, 
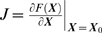
. The elements of *J* are chosen by fitting δ***X*** with corresponding experimental profiles and knowing the network topology (e.g., activation causality). Hence, the amount of response (flux propagated) along a signaling pathway can be determined using the law of mass flow conservation with *first order mass-action kinetics*, 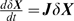 (Supplementary Table S1).

The TLR4 model begins with fixed pulse perturbation (LPS stimulation) with all other signaling processes with null activation at *t* = 0, and the elements of ***J*** are estimated knowing the network topology (i.e., activation causality) and fitting with quantitative experimental activation dynamics of key reactions, such as transcription factors' activation and target genes' induction (maintext). We considered all signaling response to be linear and sequential up to 2 hrs after LPS stimulation, therefore, parameter sensitivity in our model will not be an issue.

The *in silico* MyD88 KO and TRAF6 KO were generated from the wildtype model by setting the value of the reaction upstream(s) of MyD88 and TRAF6 respectively as null. *In silico* MyD88 overexpression was performed by increasing the rate of reaction upstream of MyD88. Supplementary Table SI lists response reactions and parameter values. The complete TLR4 model developed using E-Cell [31] is available upon request.

### Signaling Flux Conservation

The propagation of signal transduction from TLR4 to MyD88-dependent and TRAM-dependent pathways, from the law of mass conservation, becomes:
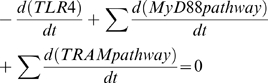
(3)where 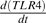 is the rate of TLR4 activation by LPS perturbation, 
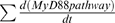
 and 
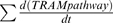
 are signaling flux of MyD88 and TRAM pathways, respectively. In *in silico* MyD88 KO, 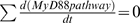 and 
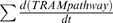
 increases, resulting in enhanced activation of the entire TRAM-dependent pathway (*SFR*). Note: We estimate the elements of Jacobian matrix by fitting with experimental data i.e. 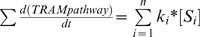, where *k_i_* is the rate constant and *S_i_* is the concentration of the *i*th activated signaling molecule in TRAM-dependent pathway consisting of *n* molecules. Hence, when 
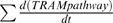
 increases, either *k_i_*, *S_i_* or both increase. In our model, for simplicity, we kept all *k_i_* fixed (except for the KO molecule between) wildtype, MyD88 KO and TRAF6 KO.

### Mice and macrophages

Nine-week-old *Myd88* ^−/−^ (MyD88 KO) mice [7] and control wildtype mice were on a C57BL/6 background. Three-week-old *Traf6* ^−/−^ (TRAF6 KO) [22] and control littermate wildtype mice backcrossed seven generations onto a C57BL/6 background were used. All mice were maintained under specific pathogen-free conditions, and experiments were performed in accordance with the Keio University guidelines for animal experimentation. M-CSF-dependent spleen-derived macrophages were prepared as described [32]. Macrophages were plated at 5×10^5^/well in a 6-well plate or 2×10^6^/well in 6 cm-dish (Falcon) and cultured in the presence of 10 ng/ml M-CSF. 100 ng/ml LPS (*Salmonella enterica* serovar Minnesota Re595, Sigma) was added to the culture media.

### Plasmids

To generate a vector expressing Glutathione S-transferase (GST) fused to the TLR4 cytoplasmic tail, a cDNA encoding mouse TLR4 (amino acids 661–835) was inserted into pME18S with GST cDNA. To generate expression vectors for Flag-TRAM and Myc-MyD88, mouse TRAM and MyD88 cDNAs were cloned into pRK5 downstream of the Flag and Myc tags, respectively.

### IRF3 immunoprecipitation and Western blot analysis

Cells were lysed for 30 min on ice in whole-cell extraction buffer (20 mM HEPES, pH 7.9, 50 mM NaCl, 1% NP-40, 2 mM EDTA, 0.5 mM EGTA, 0.5 mM phenylmethanesulfonyl fluoride, 2 mM sodium orthovanadate and proteinase inhibitors cocktail (Roche)). Lysates containing 500 mg of proteins were incubated overnight with 1 µg/ml of anti-IRF3 antibody (Santa Cruz). The immunocomplex was precipitated using protein A-Sepharose beads (R & D) for 2 h at 4°C. Beads were washed with and resuspended in SDS sample buffer, boiled for 5 min, and fractionated on polyacrylamide/SDS gel. The electrotransferred membrane was incubated with anti-serine antibody conjugated with horseradish peroxidase (Abcam), and visualized by the ECL system (Amersham Pharmacia). Whole-cell lysates were also fractionated by polyacrylamide/SDS gel electrotransferred to membranes, incubated with each antibody, and detected by ECL system. Anti-phospho-JNK, phospho-ERK, phospho-p38 antibodies were purchased from Cell Signaling. Anti-JNK, ERK, p38, and IκBα antibodies were purchased from Santa Cruz. Anti-Actin antibody is from Sigma-Aldrich.

### GST pull-down assay

HEK293T cells were cotransfected with GST-TLR4 or GST and with Flag-TRAM and Myc-MyD88 by calcium phosphate method. At 36 h after transfection, cells were harvested and lysed with TNE buffer (10 mM Tris-HCl, pH 7.5, 150 mM NaCl, 1 mM EDTA, 1% NP-40) followed by centrifugation. The supernatant was incubated with Glutathione-Sepharose beads (Amersham Pharmacia) for 30 min at 4°C. After beads were washed with TNE buffer, GST fusion protein complexes were separated on a 10% polyacrylamide/SDS gel and were transferred to a membrane. The membrane was incubated with anti-Flag M2 antibody (Sigma-Aldrich), anti-Myc 9E10 or anti-GST antibodies (Santa Cruz). The membrane was incubated with anti-rabbit or anti-mouse IgG conjugated to horseradish peroxidase, and immunoreactive proteins were visualized by the ECL system.

### Quantitative RT-PCR analysis

Total RNA was isolated from duplicated cultures in Isogen (Nippon Gene) and cDNA synthesized using the SuperScript First-Strand Synthesis System (Invitrogen). Quantitative PCR was performed on an ABI PRISM 7000 using TaqMan Assay-on-Demand (Applied Biosystems) for *Tnf*, *Socs3*, *Cxcl10* and *Ifit2*.

### Statistical analysis

Statistical significance was determined using two-tailed Student's t-test.

## Supporting Information

Table S1 In silico TLR4 model reactions and parameter values.
(0.13 MB DOC)

Figure S1 In silico simulation of NF-κB and MAP kinase activation. (A) p38, (B) ERK, (C) JNK and (D) NF-κB. Blue solid lines indicate wildtype (WT) and red dotted lines indicate MyD88 KO conditions. The x-axis represents simulation time in minutes and the y-axis represents relative activity, with the maximum value normalized to 1.
(2.17 MB TIF)

Figure S2 In silico simulation of AP-1 and NF-κB activation. (A) NF-κB, (B) JNK. Blue solid lines indicate wildtype (WT) and green dotted lines indicate MyD88 overexpressed twice WT levels. The x-axis represents simulation time in minutes and the y-axis represents relative activity, with the maximum value normalized to 1.
(1.92 MB TIF)

Figure S3 Enhanced TRAM-dependent pathway in the absence of TRAF6. (A) Ifit1 and (B) Ifit2 transcripts in wildtype (Traf6+/+) and Traf6−/− macrophages unstimulated (filled bar) or LPS (gray bar) for 60 min were analyzed by qRT-PCR and normalized to Gapdh. The values represent the average of six independent cultures and are shown as means±SEM.
(1.73 MB TIF)
