# Supplementary material for: Signaling Flux Redistribution at Toll-Like Receptor Pathway Junctions
Source: PLoS One. 2008 Oct 17;3(10):e3430. doi: 10.1371/journal.pone.0003430 (PMC2561291; doi:10.1371/journal.pone.0003430)
Supplement: Table S1 — In silico TLR4 model reactions and parameter values. (0.13 MB DOC) [file pone.0003430.s001.doc]

| **No** | **Reaction** | **Formulae** | **Parameter value (1/s)** | **Description** |
| --- | --- | --- | --- | --- |
| **MyD88-dependent Pathway** | | |  |  |
| **1** | TLR4 →MyD88/MAL | k1* TLR4 | k1= 0.017  k1= 0.0 (MyD88 KO)  k1= 0.034 (MyD88 overexpressed twice) | MyD88 and MAL are recruited to TLR4 [1,2]. We combined these processes into one signaling reaction, TLR-MyD88/MAL |
| **2** | MyD88→IRAK4/IRAK1 | k2* MyD88 | k2= 0.0017 (wildtype) | IRAK4 and IRAK1 bind to TLR-MyD88/MAL complex [2]. We combined these processes into one signaling reaction, MyD88-IRAK4/IRAK1 |
| **3** | IRAK4/IRAK1→TRAF6 | k3* IRAK | k3= 0.01 | Phosphorylated IRAKs bind to TRAF6 and are released from MyD88 |
| **4** | TRAF6→TAB/TAK | k4* TRAF6 | k4= 0.0167 | TRAF6 binds to TAB/TAK complex |
| **5** | TRAF6→ASK1 | k5* TRAF6 | k5=0.0001 | ASK1 binds to TRAF6 complex [3] |
| **6** | TAB/TAK→ IKKc | k6* TABTAK | k6= 0.0035 | Activation of MAP Kinase Kinase and IKK complex via TAB/TAK complex |
| **7** | TAB/TAK→ MKK1 | k7* TABTAK | k7= 0.00167 |
| **8** | TAB/TAK →MKK3 | k8* TABTAK | k8= 0. 00167 |
| **9** | TAB/TAK→MKK4 | k9* TABTAK | k9= 0.00167 |
| **10** | ASK1→MKK3/6 | k10* ASK1 | k10=0.001 | Activation of MKK3/6 and MKK4/7 via ASK1 [3,4] |
| **11** | ASK1→MKK4/7 | k11* ASK1 | k11=0.001 |
| **12** | IKKc→ IκBα/NF-қB | k12* IKKc | k12= 0.0167 | Phosphorylation of IκBα/NF-қB via IKK complex |
| **13** | MKK3/6→p38 | k13* MKK3 | k13= 0. 0167 | Activation of p38 via MKK3/6 |
| **14** | MKK4/7→JNK | k14* MKK4 | k14= 0. 0167 | Activation of JNK via MKK4/7 |
| **15** | MKK1/2→ERK | k15* MKK1 | k15= 0. 0167 | Activation of ERK via MKK1/2 |
| **16** | IκBα/NF-κB→ p65/p50 | k16* IκBα/NF-κB | k16= 0.0333 | Release of p65/p50 (NF-κB) after IκB phosphorylation |
| **17** | p38→p38n | k17* p38 | k17= 0.0167 | p38 translocates to the nucleus |
| **18** | JNK→ JNKn | k18* JNK | k18= 0.0167 | JNK translocates to the nucleus |
| **19** | ERK→ERKn | k19* ELK1c | k19= 0.0167 | ERK translocates to the nucleus |
| **20** | p65/p50→NF-κBn | k20* NF-κBc | k20= 0.0167 | p65/p50 (NF-κB) translocates to the nucleus |
| **21** | p38n→ AP1 | k21* p38n | k21= 0.0167 | MAP Kinases activate AP-1 |
| **22** | JNKn→ AP1 | k22* JNKn | k22= 0.00167 |
| **23** | ERKn→ AP1 | k23* ELK1n | k23= 0.0167 |
| **24** | AP1→ AP1deg | k24* AP1 | k24= 0.0022 | AP-1 degradation |
| **25** | AP1→TNFag1 | k25* AP1 | k25= 0.0007 | AP-1-DNA binding to activate *Tnf* transcription |
| **26** | NF-κBn→ Tnfag1 | k26* NF-κB | k26= 0.0003 | NF-κB -DNA binding to activate *Tnf* transcription |
| **27** | TNFag1→ TNFag2 | k27* TNFag1 | k27= 0.3 | Delay reactions due to nuclear processes leading to the transcription of *Tnf*, e.g. chromatin remodeling, transcription factor initiation and complex formation [5] |
| **28** | TNFag2→ TNFag3 | k28* TNFag2 | k28= 0.001 |
| **29** | TNFag3→ TNFa | k29* TNFag3 | k29= 0.001 | *Tnf* transcription |
| **30** | TNFa→ TNFadeg | k30* TNFα | k30= 0.00001 | *Tnf* conversion to TNF-α and *Tnf* degradation (combined) |
| **31** | AP1→Socg1 | k31* AP1 | k31= 0.0007 | AP1-DNA binding to activate *Socs3* transcription |
| **32** | NF-κBn→ Socg1 | k32* NF-κB | k32= 0.0003 | NF-κB -DNA binding to activate *Socs3* transcription |
| **33** | Socg1→ Socg2 | k33* Socg1 | k33= 0.3 | Delay reactions due to nuclear processes leading to the transcription of *Socs3,* e.g. chromatin remodeling, transcription factor initiation and complex formation |
| **34** | Socg2→ Socg3 | k34* Socg2 | k34= 0.001 |
| **35** | Socg3→ Socs3 | k35* Socg3 | k35= 0.001 | *Socs3* transcription |
| **36** | Socs3→ Socsdeg | k36* Socs3 | k36= 0.00001 | *Socs3* conversion to Socs-3 and *Socs3* degradation (combined) |
| **37** | NF-κBn→ IPG1 | k37* NF-κB | k37= 1.67e-012 | NF-κB -DNA binding to activate *Cxcl10* transcription |

| **TRAM-dependent Pathway** | | | |  |
| --- | --- | --- | --- | --- |
| **38** | TLR4→Il | k38* TLR4 | k38=0.011 |  |
| **39** | I1→ I2 | k39* I1 | k39=0.011 | Additional intermediates acting along TRAM-dependent pathways [6-8] |
| **40** | I2→I3 | k40* I2 | k40= 0.01 |
| **41** | I3→ TRAM | k41* I3 | k41= 0.01 |
| **42** | TRAM→ TRIF | k42* TRAM | k42= 0.0067 | TRIF binds to TLR-TRAM complex |
| **43** | TRIF→ TBK1 | k43* TRIF | k43= 0.00333 | TBK1 binds to TRIF at the complex |
| **44** | TBK1→ IRF3c | k44* TBK1 | k44= 0.00333 | IRF3 binds TBK1 at the complex |
| **45** | IRF3→ IRF3n | k45* IRF3 | k45= 0.000167 | IRF3 phosphorylation, release and translocation to the nucleus |
| **46** | IRF3n→ IRF3x | k46* IRF3 | k46= 0.000568 | IRF3-DNA binding to activate transcription of other genes not considered in model |
| **47** | IRF3n→ IPG2 | k47* IRF3 | k47= 0.000567 | IRF3-DNA binding to activate *Cxcl10* transcription |
| **48** | TBK1→cRel/p50/IkB | k48* TBK1 | k48= 0.0006 | Phosphorylation of cRel/p50/IkB via TBK1 |
| **49** | cRel/p50/IkB→ cRel/p50 | k49* cRel-p50/IkB | k49= 0.0001 | Release of cRel/p50 (NF-κB) after IκB phosphorylation |
| **50** | cRel/p50c→cRel/p50n | k50* cRel-p50 | k50= 0.0001 | cRel/p50 (NF-κB) translocates to the nucleus towards *Cxcl10* transcription |
| **51** | cRel/p50c→NF-κBn | k51* cRelp50c | k51= 0.099 | cRel/p50 (NF-κB) translocates to the nucleus towards *Tnf*, *Socs3* transcription |
| **52** | cRel/p50n→cRel/p50deg | k52* cRel-p50n | k52= 0.00002 | Degradation of cRel/p50 (NF-κB) |
| **53** | cRel/p50n→IPG1 | k53* cRel-p50n | k53= 0. 00001 | NF-κB -DNA binding to activate *Cxcl10* transcription |
| **54** | TRIF→ TRAF6 | k54* TRIF | k54= 0.0009 | TRIF binds to TRAF6 from the MyD88-dependent pathway |
| **55** | TRIF→ RIP1 | k55* TRIF | k55= 0.0005 | TRIF binds to RIP1, which phosphorylates AIP1, and AIP1 binds to TRAF6 [3,4] |
| **56** | RIP1→ AIP1 | k56* RIP1 | k56= 0.003 |
| **57** | AIP1→TRAF6 | k57* AIP1 | k57= 0.0033 |
| **58** | AIP1→ASK1 | k58* AIP1 | k58=0.001 | Stimulation of ASK1 by phosphorylated AIP1 [3] |
| **59** | RIP1→IKKc | k59* RIP1 | k59=0.005 | RIP1 activates IKK complex [9] |
| **60** | IPG1→ IPG2 | k60* IPG1 | k60= 0.000167 | Delay reactions due to nuclear processes leading to the transcription of *Cxcl10,* e.g. chromatin remodeling, transcription factor initiation and complex formation |
| **61** | IPG2→ IPG3 | k61* IPG2 | k61= 0.000167 |
| **62** | IPG3→ IPG4 | k62* IPG3 | k62= 0.000167 |
| **63** | IPG4→ Cxcl10 | k63* IPG4 | k63= 0.000167 | *Cxcl10* formation |
| **64** | Cxcl10→ IPdeg | k64* Cxcl10 | k64= 0.0133 | *Cxcl10* conversion to CXCL10 and *Cxcl10* degradation (combined) |

**Dealing with unknown positive or negative regulators**: Each reaction in the model and table may not be restricted only to the molecules represented. It can actually be an average representation of a few reactions’ kinetics. Therefore, unknown molecules affecting each reaction such as negative regulators are already averaged into our reactions. For example, IKKα negatively regulates NF-κB [10]. We combined the reaction IKKcomplex→IκB/(p65/p50) as a single reaction with the positive average kinetic parameter value, K = 0.0167s-1 (eq 12, Table 1). If IKKα is removed, this value will increase in our model to fit the experimental data of IKKα-deficient macrophages (data not shown). If in the case of any missing positive regulators, removal of such molecules will result in the reduction or abolishment of the overall downstream signaling kinetics, depending on how crucial the molecule is for the downstream pathway, in experiments but not in model. Using such information, we can detect both positive and negative regulators.

**References**

1. O’Neill LA (2003) The role of MyD88-like adapters in Toll-like receptor signal transduction. Biochem Soc 31: 643- 647.

2. Horng T, Barton G, Flavell R, Medzhitov R (2002) The adaptor molecule TIRAP provides signalling specificity for Toll-like receptors. Nature 420: 329-333.

3. Matsuzawa A, Saegusa K, Noguchi T, Sadamitsu C, Nishitoh H, Nagai S, Koyasu S, Matsumoto k, Takeda K, Ichijo H (2005) ROS-dependent activation of the TRAF6-ASK1-p38 pathway is selectively required for TLR4-mediated innate immunity. Nat Immunol 6: 587-592.

4. Zhang H, Lin Y, Li J, Pober JS, Min W (2007) **RIP1-mediated AIP1 phosphorylation at a 14-3-3-binding site is critical for tumor necrosis factor-induced ASK1-JNK/p38 activation.** J Biol Chem. 282: **14788-14796.**

5. Smale ST, Fisher AG (2002) Chromatin structure and gene regulation in the immune system. Annu. Rev. Immunol. 20: 427-462.

6. Selvarajoo K (2006) Discovering differential activation machinery of the Toll-like receptor 4 signaling pathways in MyD88 knockouts. FEBS Lett. 580: 1457-1464.

7. McGettrick AF, Brint EK, Palsson-McDermott EM, Rowe DC, Golenbock DT, Gay NJ, Fitzgerald KA, O'Neill LAJ (2006) Trif-related adapter molecule is phosphorylated by PKCε during Toll-like receptor 4 signaling. Proc Natl Acad Sci U S A 103: 9196-9201.

8. Kagan JC, Su T, Horng T, Chow A, Akira S, Medzhitov R (2008) TRAM couples endocytosis of Toll-like receptor 4 to the induction of interferon-β. Nat Immunol 9: 361 – 368.

9. Cusson-Hermance N, Khurana S, Lee TH, Fitzgerald KA, Kelliher MA. et al. (2005) Rip1 mediates the Trif-dependent toll-like receptor 3 and 4-induced NF-κB activation but does not contribute to IRF-3 activation. J Biol Chem. 280: **36560-36566.**

10. Lawrence T, Bebien M, Liu GY, Nizet V, Karin M (2005) IKKα limits macrophage NF-κB activation and contributes to the resolution of inflammation. Nature 434: 1138-43.
