# Supplementary figures and images for: Signaling Flux Redistribution at Toll-Like Receptor Pathway Junctions
Source: PLoS One. 2008 Oct 17;3(10):e3430. doi: 10.1371/journal.pone.0003430 (PMC2561291; doi:10.1371/journal.pone.0003430)

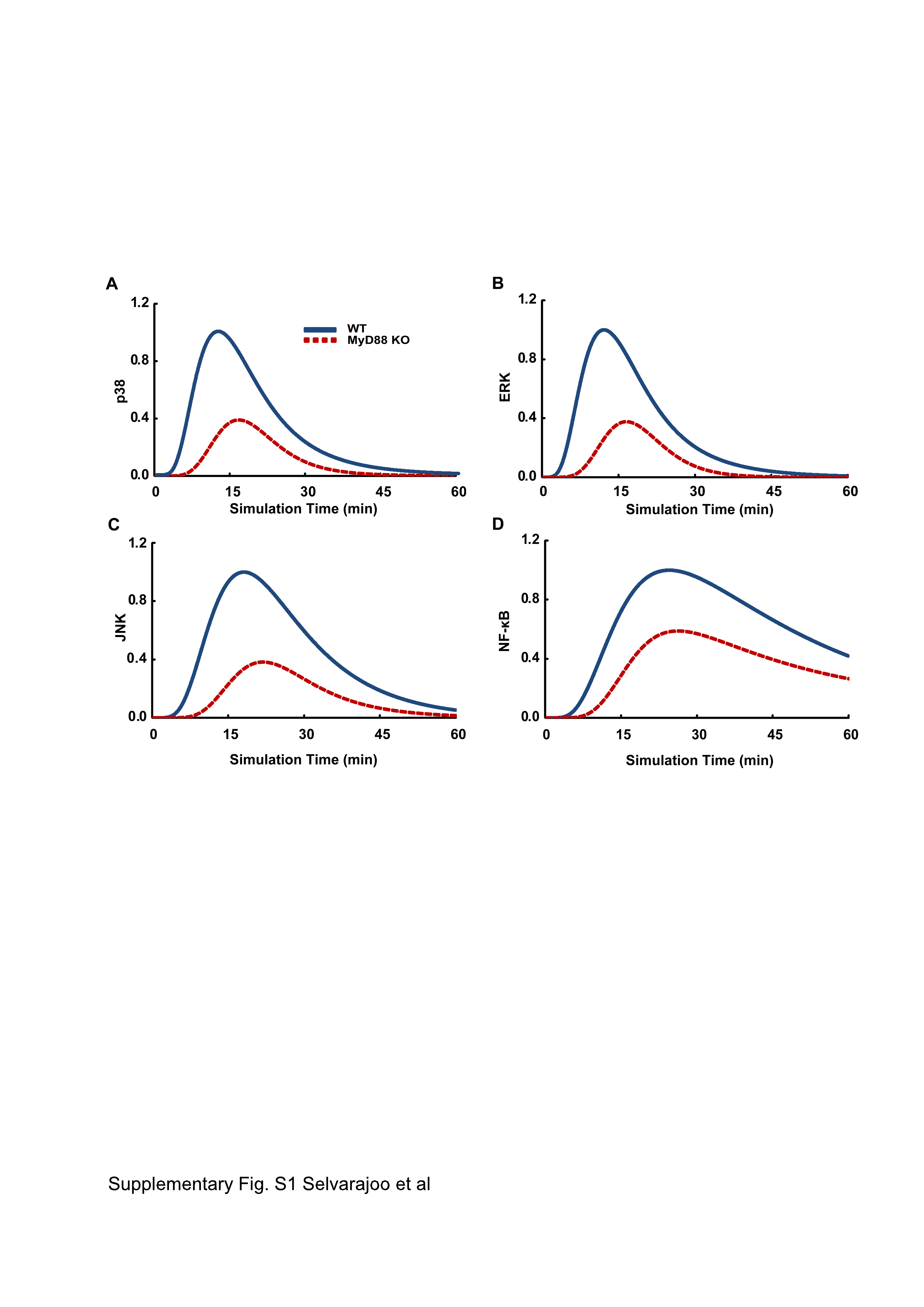

Supplement: Figure S1 — In silico simulation of NF-κB and MAP kinase activation. (A) p38, (B) ERK, (C) JNK and (D) NF-κB. Blue solid lines indicate wildtype (WT) and red dotted lines indicate MyD88 KO conditions. The x-axis represents simulation time in minutes and the y-axis represents relative activity, with the maximum value normalized to 1. (2.17 MB TIF) [file pone.0003430.s002.tif]

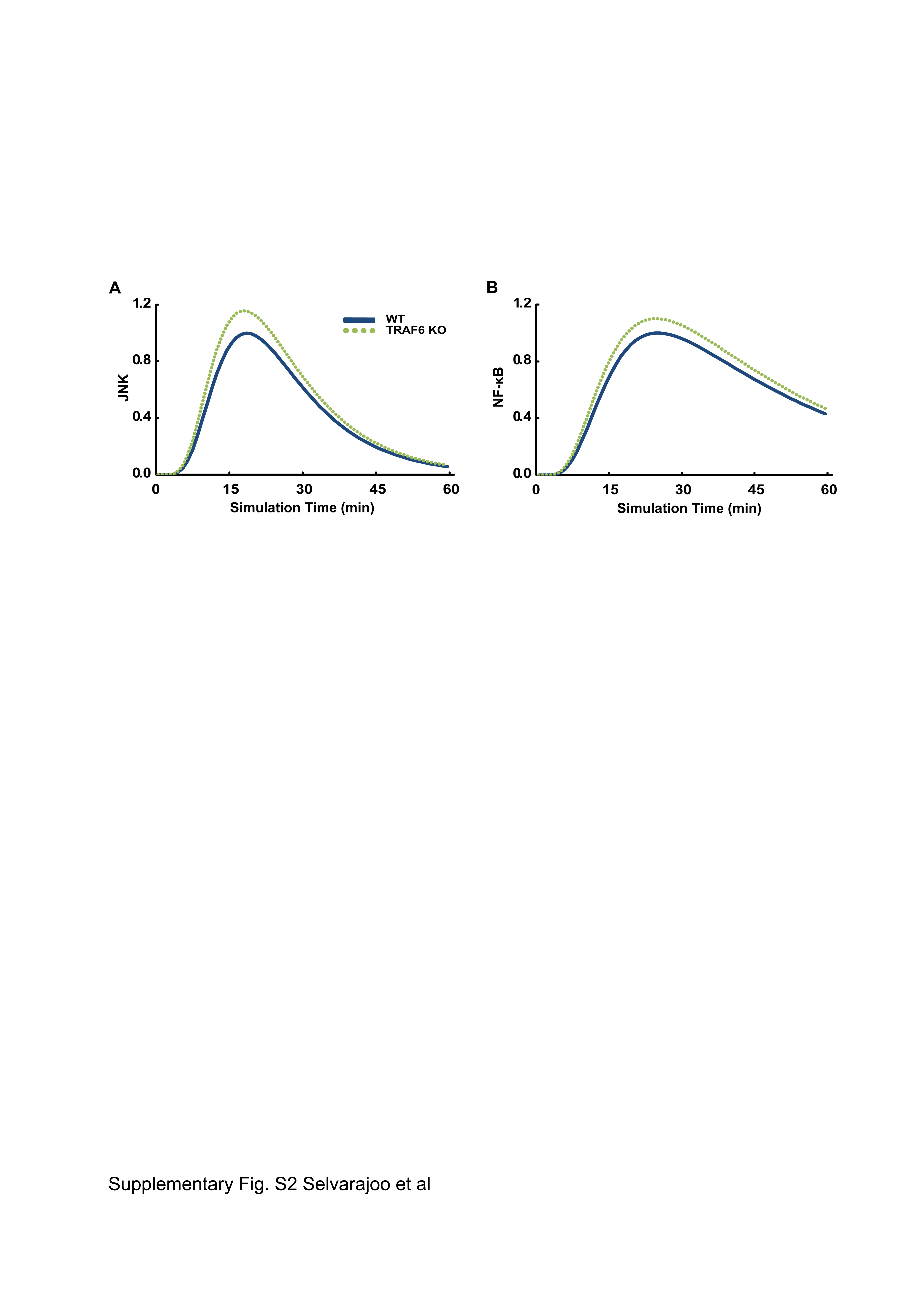

Supplement: Figure S2 — In silico simulation of AP-1 and NF-κB activation. (A) NF-κB, (B) JNK. Blue solid lines indicate wildtype (WT) and green dotted lines indicate MyD88 overexpressed twice WT levels. The x-axis represents simulation time in minutes and the y-axis represents relative activity, with the maximum value normalized to 1. (1.92 MB TIF) [file pone.0003430.s003.tif]

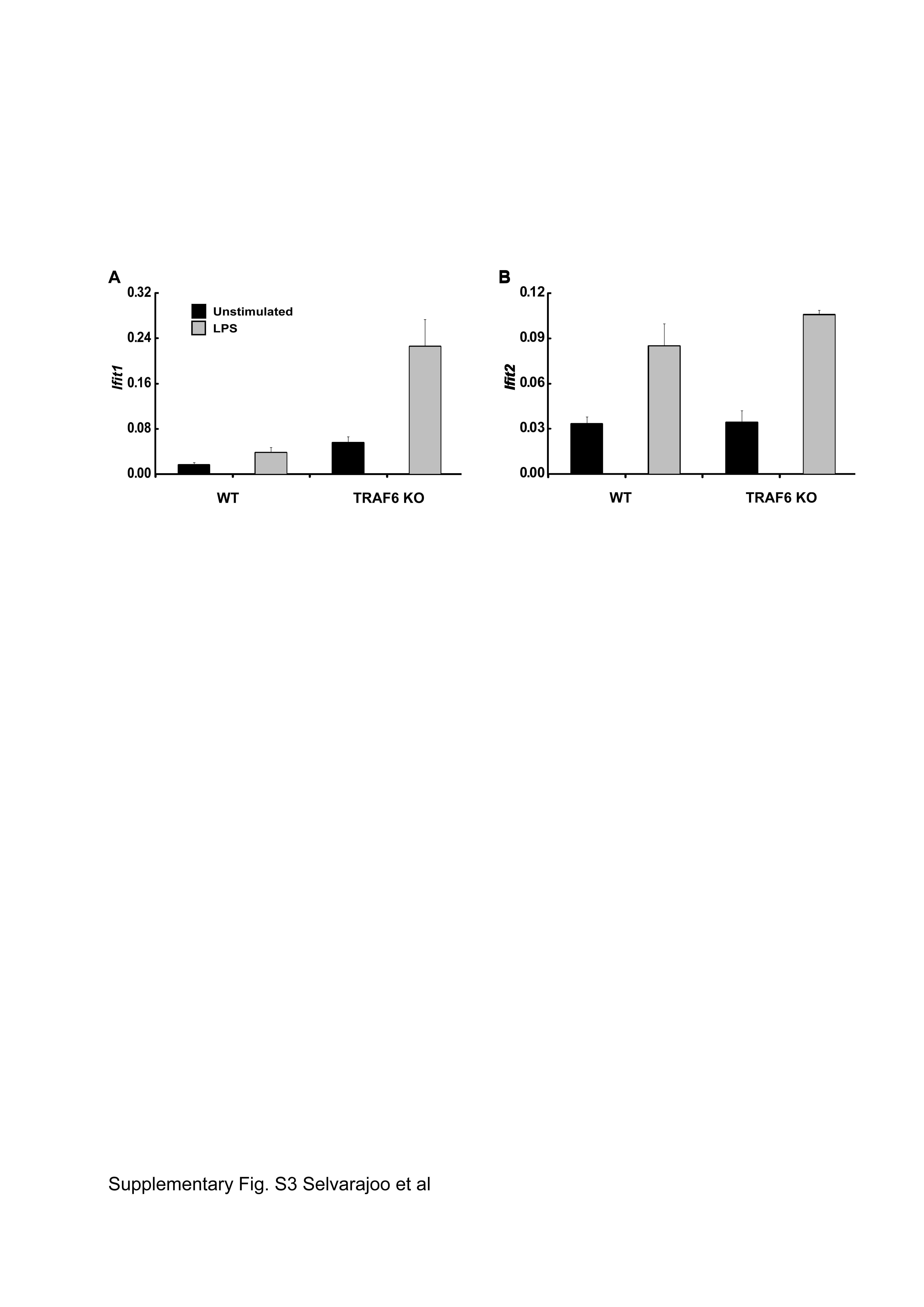

Supplement: Figure S3 — Enhanced TRAM-dependent pathway in the absence of TRAF6. (A) Ifit1 and (B) Ifit2 transcripts in wildtype (Traf6+/+) and Traf6−/− macrophages unstimulated (filled bar) or LPS (gray bar) for 60 min were analyzed by qRT-PCR and normalized to Gapdh. The values represent the average of six independent cultures and are shown as means±SEM. (1.73 MB TIF) [file pone.0003430.s004.tif]
